# Testosterone Levels and Type 2 Diabetes—No Correlation with Age, Differential Predictive Value in Men and Women

**DOI:** 10.3390/biom8030076

**Published:** 2018-08-20

**Authors:** Mahir Karakas, Sarina Schäfer, Sebastian Appelbaum, Francisco Ojeda, Kari Kuulasmaa, Burkhard Brückmann, Filip Berisha, Benedikt Schulte-Steinberg, Pekka Jousilahti, Stefan Blankenberg, Tarja Palosaari, Veikko Salomaa, Tanja Zeller

**Affiliations:** 1 Department of General and Interventional Cardiology, University Heart Center, 20246 Hamburg, Germany; m.karakas@uke.de (M.K.); sar.schaefer@uke.de (S.S.); sebastian.appelbaum@tu-dortmund.de (S.A.); f.ojeda-echevarria@uke.de (F.O.); Burkhard.Brueckmann@uk-erlangen.de (B.B.); f.berisha@uke.de (F.B.); b.schulte-steinberg@uke.de (B.S.-S.); s.blankenberg@uke.de (S.B.); 2 German Center for Cardiovascular Research (DZHK), Partner Site Hamburg, 20246 Hamburg, Germany; 3 National Institute for Health and Welfare, 00271 Helsinki, Finland; kari.kuulasmaa@thl.fi (K.K.); pekka.jousilahti@thl.fi (P.J.); tarja.palosaari@thl.fi (T.P.); veikko.salomaa@thl.fi (V.S.)

**Keywords:** testosterone, diabetes, age, prognosis, men, women

## Abstract

Most studies reporting on the association of circulating testosterone levels with type 2 diabetes in men are of cross-sectional design. Reports on the relevance of altered testosterone levels in women are scarce. Here, we evaluate the role of low serum testosterone levels for incident diabetes in men and women in a population setting of 7706 subjects (3896 females). During a mean follow up time of 13.8 years, 7.8% developed type 2 diabetes. Significant correlations of testosterone with high density lipoprotein (HDL)-cholesterol (*R* = 0.21, *p* < 0.001), body-mass-index (*R* = −0.23, *p* < 0.001), and waist-to-hip-ratio (*R* = −0.21, *p* < 0.001) were found in men. No correlation was found with age in men; in women, the correlation was negligible (*R* = 0.04, *p* = 0.012). In men, low testosterone levels predicted high risk of type 2 diabetes, while in women this relationship was opposite. Men with low testosterone levels showed increased risk of future diabetes (hazard ratio (HR) 2.66, 95% confidence interval (CI) 1.91–3.72, *p* < 0.001 in basic model; HR 1.56 95%, CI 1.10–2.21, *p* = 0.003). In women, low testosterone levels indicated lower risk with (HR 0.53, 95% CI 0.37–0.77, *p* = 0.003), while the association lost significance in the fully adjusted model (HR 0.72, 95% CI 0.49–1.05, *p* = 0.09). Low levels of testosterone predicted future diabetes in men. A borderline opposite association was found in women.

## 1. Introduction

Testosterone is the primary male sex hormone and an anabolic steroid, and it seems widely accepted that men experience a gradual decline in testosterone levels with increasing age [1,2,3,4]. The potential association of circulating testosterone with diabetes is of utmost clinical interest: Since the Endocrine Society addressed the high prevalence of low serum testosterone levels in patients with type 2 diabetes in its Clinical Practical Guidelines of 2010, testosterone has moved into the focus of cardiometabolic therapy, and its prescription escalated at startling rates creating a $2 billion annual market in the U.S. [5,6]. Importantly, no randomized clinical trial has provided proof of the benefit of testosterone therapy. Recently, the testosterone lowering properties of statins have come into focus [7]. Large meta-analyses of randomized clinical trials have demonstrated an increased risk of new-onset diabetes with statin therapy [8,9,10,11]. Statins lower testosterone levels by their very own property, as cholesterol is a precursor of the testosterone biosynthesis pathway, but also by a selective inhibitory effect on 17-ketosteroid-oxidoreductase enzyme activity [12,13]. Given the fact that almost 20 million Europeans are taking statins, and considering that the percentage of U.S.-American adults aged ≥40 taking statins rose from 18 to 26% between 2003 and 2012, the impact of these findings is tremendous [14]. 

Since most studies reporting on the association of low circulating testosterone levels with type 2 diabetes in men are of cross-sectional design, and since reports on the relevance of altered testosterone levels in women are scarce, the clinical relevance of low testosterone levels remains unclear [15,16,17,18].

In a large population-based European cohort study, we aimed (i) to assess the distribution of testosterone values and compare it to current references, (ii) to evaluate the correlation of testosterone levels with age and major clinical variables, and (iii) to prospectively evaluate the potential role of low serum testosterone levels for incident diabetes in men and women.

## 2. Materials and Methods

### 2.1. Study Population

The FINRISK study is a prospective population-based study carried out in five districts in Finland, i.e., North Karelia, Northern Savo (former Kuopio Province), Southwestern Finland, Oulu Province, and the region of Helsinki and Vantaa. The design of the study has been published elsewhere [19]. Of the 11,500 subjects, aged 25–74 years, that were invited to participate in the study, 8444 subjects (73%) participated in the clinical examination. The follow-up period was up to 15 years. Individuals that participated in the study received a physical examination, a blood sample was drawn, and they filled in a self-administered questionnaire. Prior to the study participation, individuals were asked for a 4 h fasting period and to avoid heavy meals during the day. The median fasting time was 5 h, with an interquartile range of 3–7 h. The blood samples were processed and stored at −80 °C. For the present paper, pregnant women, individuals on testosterone-supplementation therapy, and individuals with prevalent cancer or diabetes were excluded. Therefore, final analyses were based on 7706 individuals (49.4% men and 50.6% women). The Ethics Committee of the National Public Health Institute approved the study, which followed the Declaration of Helsinki. The study was approved by the Ethics Committee of the National Public Health Institute, Helsinki (82/2001). All subjects gave written informed consent.

### 2.2. Outcome Information

To identify subjects that developed incident diabetes during follow-up, the National Hospital Discharge Register, the National Causes of Death Register and the National Drug Reimbursement Register were used. The definition of the endpoint diabetes mellitus was based on (i) the drug reimbursement records of hypoglycemic drugs (ATC-class A10) from the Drug Reimbursement Register SII, (ii) the records of patients entitled to fully reimbursed diabetes medication, (iii) the National Hospital Discharge Register for hospitalizations with diabetes (E10-E14/ICD-10; 250/ICD-9), and (iv) the National Causes-of-Death Register with diabetes (ICD-codes as above) as the underlying, direct, or contributing cause of death. The validity of Finnish national health care registries for identifying disease outcomes has recently been shown [20,21].

### 2.3. Laboratory Methods

Blood samples were stored at −80 °C under standardized conditions. Routine laboratory parameters were determined at the Disease Risk Unit in the National Institute for Health and Welfare, Helsinki, Finland [19]. The estimated glomerular filtration rate (eGFR) was derived using the CKD-Epi equation based on creatinine [22]. Total testosterone levels were measured in serum samples using a chemiluminescent microparticle immunoassay (CMIA) (Abbott ARCHITECT 2nd Generation Testosterone; Abbott Diagnostics, Wiesbaden, Germany) at the BiomarCaRE Laboratory (University Heart Center Hamburg, Germany) [23]. The assay range was 0.45–35 nmol/L, the interassay coefficient of variation (CV) was 8.83%, the intra-assay CV was 3.57%.

### 2.4. Statistical Methods

Multiple imputation via chained equations were used to handle missing values [24]. Baseline characteristics are presented as median and interquartile ranges (IQRs) for continuous variables and as counts and percentages for dichotomous variables. Pearson correlation coefficients and age-adjusted Pearson correlations were used to perform correlation analysis. Using categorized (by sex specific quartiles) testosterone levels age-adjusted Kaplan-Meier curves for incident type 2 diabetes were generated. Cox regression models were used to examine the association of testosterone with type 2 diabetes, using age as the time scale and were performed with testosterone as a continuous and a categorized variable (using sex-specific quartiles). Due to the different shape of the testosterone distributions in men and women, each sex was considered separately. Two different adjustments were used: (i) age as the time-scale, and adjusted for geographical region of Finland (east, west) (Model 1) and (ii) Model 1 and additional adjustment for log-transformed high density lipoprotein (HDL)-cholesterol, smoking status, total cholesterol, log-transformed systolic blood pressure, waist-hip-ratio, and time of blood draw, since testosterone values undergo a circadian rhythm (Model 2). For all analyses, the R version 3.2.2 (R Foundation for Statistical Computing, Vienna, Austria). All tests were two-tailed, and *p* < 0.05 was considered statistically significant.

## 3. Results

The baseline characteristics of the study participants are presented in Table 1. The mean age of the participants was 49.0 years for men and 47.0 years for women. Men in general had a more adverse cardiovascular risk profile than women. As shown in Table S1 and Figure S1 in the Supplement, testosterone levels were clearly higher in male subjects (median 17.12 versus 1.15 nmol/L; *p* < 0.001).

In order to assess the correlation of testosterone levels with clinical variables, Pearson correlation coefficients were calculated (Table 2). In crude Pearson analyses, no correlation was found with age in men (*R* = 0.02; *p* = 0.19), while correlation was negligible in women (*R* = 0.04; *p* = 0.012). Age-adjusted Pearson analyses revealed statistically significant correlations of testosterone levels with HDL-cholesterol levels (*R* = 0.21, *p* < 0.001), body-mass-index (BMI) (*R* = −0.23; *p* < 0.001), and waist-to-hip-ratio (*R* = −0.21; *p* < 0.001) in men. In line, all other nominal correlations in women—with eGFR (*R* = −0.05; *p* = 0.007) and systolic blood pressure (*R* = 0.04; *p* = 0.046)—were also negligible (*R* ≤ ±0.05).

**Figure 1:**
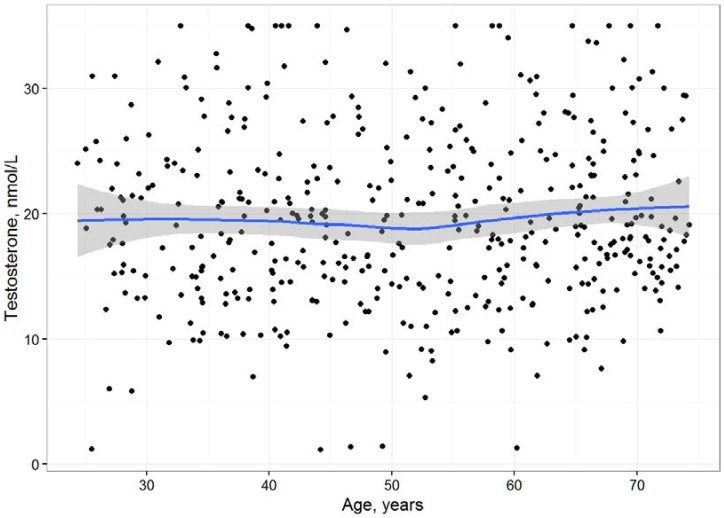
Correlation (*R* = 0.02, *p* = 0.62) between age and testosterone (analysis restricted to those men with blood drawn before noon).

As shown in Figure 1, correlation analysis between testosterone and age, limited to those men with blood drawn before noon, also did not show any significant correlation (*R* = 0.02, *p* = 0.62). Adjustment of this correlation for waist-to-hip-ratio even revealed a positive correlation between age and testosterone (*R* = 0.15, *p* = 0.001).

**Figure 2:**
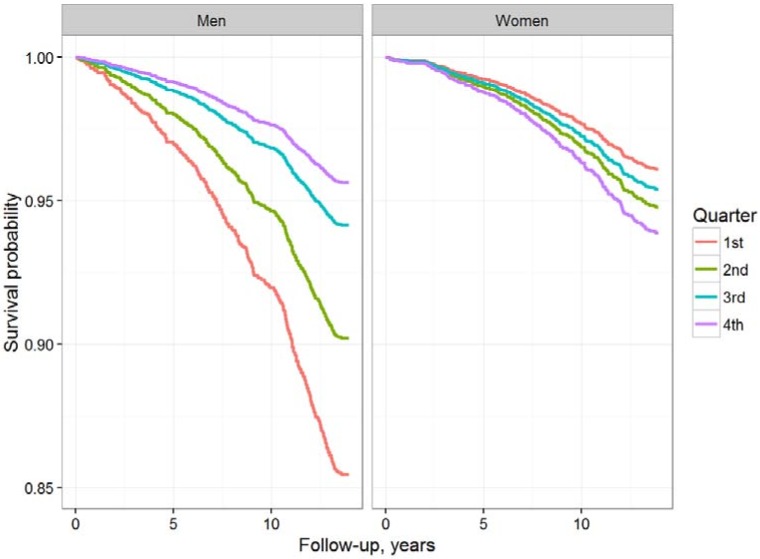
Age-adjusted Kaplan-Meier-Curves (men and women) according to sex-specific testosterone categories for incident type 2 diabetes during follow-up.

During a median follow-up of 13.8 years, a total of 599 incident type 2 diabetes cases (7.8%) were identified (363 male, 236 female). In men, Kaplan-Meier analyses showed a strong association of low testosterone levels with incident diabetes, while women at higher testosterone quarters were at higher risk for future type 2 diabetes (Figure 2). In accordance, basic (model 1) and fully adjusted (model 2) Cox regression analyses in men indicated 56% higher hazard of future diabetes in those with low testosterone levels (hazard ratio (HR) 1.56, 95% confidence interval (CI) 1.10–2.21, *p* = 0.01 for highest versus lowest quarter in the fully adjusted model) (Table 3). In women, basic adjusted Cox regression analysis, where age was used as the time scale, and only geographical region was adjusted for, indicated lower risk with low testosterone levels (HR 0.53, 95% CI 0.37–0.77, *p* = 0.001 for highest versus lowest quarter). This association lost its significance and became borderline non-significant upon additional adjustment for conventional cardiovascular risk factors, waist-hip-ratio, and time of blood draw (HR 0.72, 95% CI 0.49–1.05, *p* = 0.09 for highest versus lowest quarter). 

Table 4 shows the percentage of subjects stratified for three age groups and respective testosterone levels and the rate of incident diabetes. As seen in the table, and also evidenced by age- and sex-adjusted Cox regression analyses, the association between testosterone and incident diabetes seems robust across different age groups.

## 4. Discussion

In this study, we evaluated the predictive value of serum testosterone levels for the incidence of type 2 diabetes in men and women.

We report three major findings: first, with median testosterone levels of 17.12 nmol/L in men, and 1.15 in women, our measurements seem in range with current references, like the Mayo medical laboratories standard (reference values in men: <age 50: 14.8–58.9 nmol/L; age 50–70: 12.0–54.1 nmol/L; >age 70: 11.4–42.3) (reference values in women: <age 50: 0.21–3.19 nmol/L; age 50–70: 0.21–2.91 nmol/L; >age 70: 0.21–2.64). Accordingly, only the minority of our population would classify as testosterone-deficient.

Secondly, levels of testosterone were clearly inversely correlated with body-mass-index and waist-to-hip-ratio (in men), while correlations with conventional cardiovascular risk factors were negligible, if significant at all. Moreover, no correlation was found with age in men (*R* = 0.02; *p* = 0.19), while correlation was negligible in women (*R* = 0.04; *p* = 0.012).

Thirdly, the current data indicates an association between low testosterone levels and future risk of type 2 diabetes in men, even after adjustment for waist-to-hip-ratio, while for women, in the fully adjusted Cox regression model, an association could not be confirmed. Of special interest: this finding surprisingly demonstrates that testosterone levels, even at lower levels of the normal range, may affect risk for future type 2 diabetes.

### 4.1. Circulating Levels of Total Testosterone

Total testosterone levels of the National Health and Nutrition Examination Survey (NHANES), were recently reported [25]. The authors reported values for 3327 female and 3419 male participants of all ages and races. In non-Hispanic white men, median testosterone level was 12.6 nmol/L, with IQR of 8.6 and 17.1 nmol/L, thereby lower than in our population (median 17.12 nmol/L, IQR 12.91–22.02). Excluding those aged 6 to 19 still resulted in lower medians than in our population. In non-Hispanic white women, median testosterone level was 0.7 nmol/L, with IQR of 0.4 and 0.9 nmol/L, thereby again lower than in our population (median 1.15 nmol/L, IQR 0.87–1.56). In this context, it is important to highlight the differences between both populations: in contrast to our population, in NHANES pregnant women, individuals on testosterone-supplementation therapy, and individuals with prevalent cancer or diabetes were not excluded. Moreover, participants were not asked for a fasting period before blood draw. These differences might explain why our levels are in line with Mayo medical laboratories references, but do not comply with NHANES data.

### 4.2. Correlation of Testosterone with Age

In our study, age did not hamper the disease association, and did not show any significant correlation with testosterone levels, even when correlation analysis was limited to those men with blood drawn before noon. Multiple previous studies indicated that serum testosterone appears to decline as men age [2,3]. Although the described decline was usually modest, circulating levels were shown to fall below the normal range of healthy young men, in whom the reference values were determined [4]. In contrast, moderate but statistically significant inverse correlations of testosterone levels in our study were seen for body-mass-index and waist-to-hip-ratio. However, these correlations were not observed in women, which presented with clearly lower testosterone levels. The negative correlation with body-mass-index/obesity and waist-to-hip-ratio/increased abdominal adiposity in men is most probably explained by elevated aromatase levels, which lower the availability of pituitary gonadotrophins, and activate the conversion of testosterone to estradiol [26]. Most previous studies reporting on the association with diabetes did not adjust for body-mass-index or waist-to-hip-ratio.

### 4.3. Association of Testosterone with Incident Type 2 Diabetes and Its Clinical Relevance

Just like coronary heart disease, type 2 diabetes is a complex epidemic disease [27,28,29,30]. The Endocrine Society addressed the high prevalence of low serum testosterone levels in patients with type 2 diabetes in its 2010 Clinical Practical Guidelines [31]. In 2014, around 2.3 million men in the U.S. were under testosterone supplementation [32]. Currently, there is a strong ongoing debate regarding potential risk and benefits of testosterone supplementation therapy [5]. Both large regulatory authorities in medicine—Food and Drug Administration (FDA), and European Medicines Agency (EMA) launched an urgent call for more high-quality clinical data on the testosterone hypothesis [32]. While multiple cross-sectional analyses gave conflicting results, and meta-analyses seemed to overestimate the potential association, our results reflect strict adjustment for covariates, which affect cardiometabolic risk and are minimally, but independently, associated with testosterone status. In both, Kaplan-Meier analyses and basic Cox regression analyses, low testosterone levels showed differential predictive value in men and women, while in women, the Cox regression-based association lost its significance upon additional adjustment for conventional cardiovascular risk factors, waist-hip-ratio, BMI, and time of blood draw (HR 0.72, 95% CI 0.49–1.05, *p* = 0.09 for highest versus lowest quartile). One may hypothesize, that loss of association in the fully adjusted model in women might be due to the smaller number of incident cases, and might gain significance in a larger cohort. This finding clearly demonstrates the relevance of testosterone in women. Of interest, while men have higher circulating levels of testosterone, it is the most abundant active sex steroid in women throughout the female lifespan [33]. Interestingly, also the correlation with BMI and WHR is sex-specific, and not relevant in women. The negative correlation between body-mass-index, waist-to-hip-ratio and circulating testosterone levels in women seems unclear, but in men seems to be explained by enhanced conversion of androstenedione to estrogens in obese males by aromatization, which occurs due to adipose tissue-driven elevated aromatase levels [34]. Moreover, studies in rodents and humans documented that metabolic endotoxemia leads to a decline in gonadal function, and that leptin, produced by adipose tissue, inhibits testosterone secretion from the Leydig cells [35,36,37].

### 4.4. Strengths and Limitations

The present study is one of the largest prospective studies investigating testosterone levels and incident diabetes; additionally, it includes women. There are remaining questions for future studies: first, we are not able to examine the effect of changes in testosterone levels towards future disease development as no serial testosterone measurements are available in our study. Second, no information is available on intra-individual increasing (for example by supplementation therapy) or decreasing testosterone levels (for example by statin intake), and we are not able to comment on whether these changes would impact future risk of diabetes. Further, we adjusted the Cox regression analyses for time of blood drawn in model 2 (although linear correlation with testosterone was negligible; Table 2), as testosterone exerts a diurnal variation, and blood samples collected in our study were drawn throughout the day. Furthermore, age was truncated, since our study included subjects aged 25–74. There might be a correlation between advanced age (above/equal 75 years) and testosterone which we did not detect. Levels of sex hormone-binding globulin (SHBG), as well as free testosterone, were not measured in the present study, which is of relevance since SHBG was shown to predict incident diabetes in men [38]. Commonly, bioavailable and free testosterone levels parallel those of total testosterone levels. However, several rare conditions have been shown to change SHBG levels. This change may cause total testosterone levels to change without influencing the bioavailable and/or free levels of testosterone. Nevertheless, the determination of total testosterone levels is consistent with clinical reality and with the recommendations of the Food and Drug Administration, which urges to measure total testosterone only. Moreover, we have no information on the use of glucocorticoids at baseline or during follow-up. This is a limiting factor, since glucocorticoids represent a common cause of drug-induced hypogonadism and type 2 diabetes. Finally, blood samples were stored over a long time period and degradation processes that might affect testosterone levels cannot be excluded.

## 5. Conclusions

In our large-scale primary-prevention study, low levels of testosterone at baseline predicted future diabetes risk, although differentially in men and women. Given the relevant correlations with body-mass-index and waist-to-hip-ratio, it may be more prudent to aim at reducing excess abdominal fat, rather than treating testosterone to a specific level.

## Figures and Tables

**Table 1:** Characteristics of study cohort at baseline.

	Men	Women
***n***	3810	3896
Age (years)	49.0 (37.5; 60.6)	47.0 (36.4; 57.5)
BMI (kg/m²)	26.5 (24.2; 29.0)	25.4 (22.7; 28.8)
Waist-hip-ratio	0.93 (0.88; 0.97)	0.79 (0.75; 0.84)
Current smoker (%)	26.5	17.5
Hypertension medication (%)	16.9	13.8
HDL-C (mmol/L)	1.23 (1.04; 1.43)	1.51 (1.28; 1.75)
Total-Cholesterol (mmol/L)	5.4 (4.8; 6.2)	5.4 (4.7; 6.1)
Systolic blood pressure (mmHg)	137 (126; 151)	129 (117; 145)
Testosterone (nmol/L)	17.12 (12.91; 22.02)	1.15 (0.87; 1.56)

BMI = body mass index, HDL-C = high-density lipoprotein-cholesterol, T2D = type 2 diabetes mellitus; For continuous variables, median (25th percentile; 75th percentile) are shown. For binary variables percentage is given.

**Table 2:** Age-adjusted Pearson correlation coefficients of testosterone levels with clinical variables.

Clinical Variable	Men	Women
Time of day of the blood draw	−0.11 <0.001	−0.03 <0.001
Age (crude analysis)	0.02 0.19	0.04 0.012
Smoking	0.09 <0.001	−0.01 0.69
Total cholesterol	0.009 0.98	−0.01 0.43
HDL-C	0.21 <0.001	−0.03 0.052
Systolic Blood Pressure	−0.04 0.025	0.04 0.046
eGFR	0.009 0.96	−0.05 0.007
BMI	−0.23 <0.001	0.03 0.13
WHR	−0.21 <0.001	0.03 0.098

BMI = body mass index, HDL-C = high-density lipoprotein-cholesterol, T2D = type 2 diabetes mellitus; eGFR = estimated glomerular filtration rate, WHR = waist-to-hip-ratio. For continuous variables, median (25th percentile; 75th percentile) are shown. For binary variables percentage is given.

**Table 3:** Association of baseline testosterone levels with type incident 2 diabetes (HR (95% CI)).

	Quarter 1 (Highest)	Quarter 2	Quarter 3	Quarter 4 (Lowest)	*p*-Value (Highest vs. Lowest Quarter)	*p*-Value for Trend
Men						
Model 1	1	1.19 (0.80–1.76)	1.93 (1.37–2.73)	2.66 (1.91–3.72)	<0.001	<0.001
Model 2	1	0.99 (0.67–1.48)	1.33 (0.94–1.89)	1.56 (1.10–2.21)	0.003	0.005
Women						
Model 1	1	0.66 (0.46–0.95)	0.77 (0.55–1.08)	0.53 (0.37–0.77)	0.003	0.003
Model 2	1	0.80 (0.55–1.16)	0.98 (0.69–1.38)	0.72 (0.49–1.05)	0.090	0.200

HR = hazard ratio, CI = confidence interval. Model 1: age used as time-scale, adjusted for geographical region. Model 2: additionally adjusted for total cholesterol (log), HDL-cholesterol (log), systolic blood pressure (log), known hypertension, smoking status, waist-hip-ratio, and time of day of the blood draw.

**Table 4:** Development of type 2 diabetes stratified for age and sex (HR (95% CI)).

	Testosterone Quarters 1–3	Testosterone Quarter 4 (Lowest)	*p*-Value
Men			
Individuals <50 years developing T2DM	70/1486 (3.5%)	39/525 (1.9%)	0.90
Individuals 506–5 years developing T2DM	115/879 (10.0%)	67/278 (5.8%)	1.00
Individuals >65 years developing T2DM	44/492 (6.9%)	28/150 (4.3%)	0.99
Women			
Individuals <50 years developing T2DM	56/1738 (2.5%)	9/520 (0.4%)	0.76
Individuals 506–5 years developing T2DM	89/898 (6.8%)	31/397 (2.4%)	0.60
Individuals >65 years developing T2DM	45/272 (13.1%)	6/71 (1.8%)	0.78

T2DM = type 2 diabetes.

## Supplementary Materials

The following are available online at http://www.mdpi.com/2218-273X/8/3/76/s1, Table S1: Distribution of baseline testosterone levels according to sex (nmol/L), Figure S1: Distribution of baseline testosterone levels according to sex. (A,B) are shown for absolute testosterone levels, (C,D) are shown for log transformed testosterone levels.
