# Supplementary material for: Testosterone Levels and Type 2 Diabetes—No Correlation with Age, Differential Predictive Value in Men and Women
Source: Biomolecules. 2018 Aug 20;8(3):76. doi: 10.3390/biom8030076 (PMC6165105; doi:10.3390/biom8030076)

Supplementary Material

Testosterone Levels and Type 2 Diabetes–

No Correlation with Age, Differential Predictive Value in Men and Women

Running Title: Testosterone levels and Type 2 Diabetes

Mahir Karakas[1,2], MD; Sarina Schäfer[1,2], MD; Sebastian Appelbaum[1], MSc; Francisco Ojeda[1], PhD; Kari Kuulasmaa[3], PhD; Burkhard-Ekkehart Brueckmann[1]; Filip Berisha[1], MD; Benedict Schulte-Steinberg[1], MD; Pekka Jousilahti[3], MD; Stefan Blankenberg[1,2], MD; Tarja Palosaari[3], MSc; Veikko Salomaa[3], MD and Tanja Zeller[1,2*], PhD

[1] Department of General and Interventional Cardiology, University Heart Center, 20246 Hamburg, Germany, m.karakas@uke.de, sar.schaefer@uke.de, sebastian.appelbaum@tu-dortmund.de, f.ojeda-echevarria@uke.de, Burkhard.Brueckmann@uk-erlangen.de, f.berisha@uke.de, b.schulte-steinberg@uke.de, s.blankenberg@uke.de, t.zeller@uke.de

[2] German Center for Cardiovascular Research (DZHK), Partner Site Hamburg, Lübeck, Kiel, Hamburg, 20246 Hamburg Germany, m.karakas@uke.de, sar.schaefer@uke.de, s.blankenberg@uke.de, t.zeller@uke.de

[3] National Institute for Health and Welfare, 00271Helsinki, Finland, kari.kuulasmaa@thl.fi, pekka.jousilahti@thl.fi, tarja.palosaari@thl.fi, veikko.salomaa@thl.fi

Table S1.

Distribution of baseline testosterone levels according to sex [nmol/L]

	Quartile 1	Quartile 2	Quartile 3	Quartile 4
Men, IQR	0; 12.91	12.91; 17.12	17.12; 22.02	22.02; 35.0
Women, IQR	0; 0.87	0.87; 1.15	1.15; 1.56	1.56; 35.0

Values are shown as normalized median values; IQR = interquartile range

Figure S1

Distribution of baseline testosterone levels according to sex. A and B are shown for absolute testosterone levels, C and D are shown for log transformed testosterone levels.

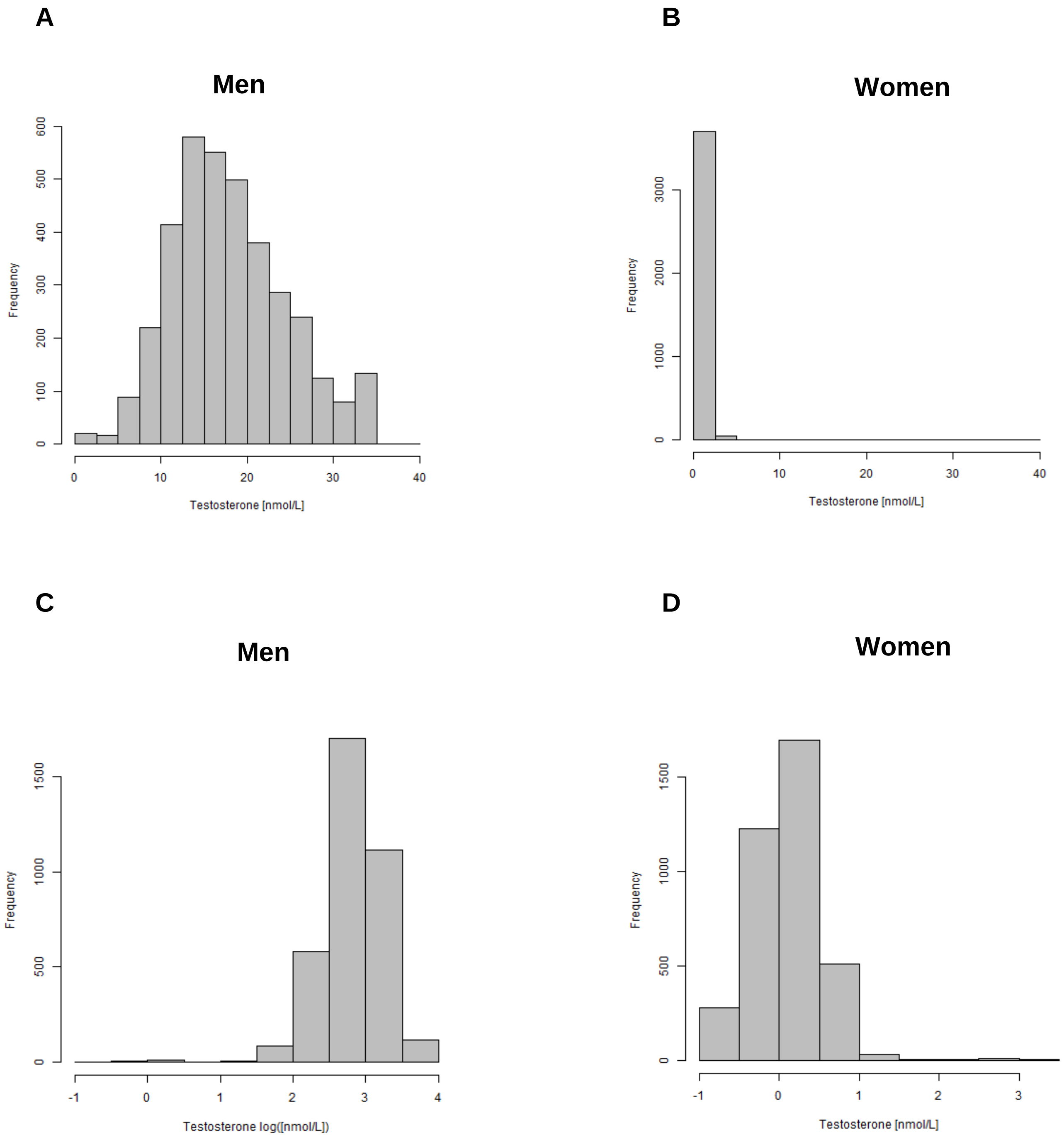

Supplement: Supplementary file 1 [file biomolecules-08-00076-s001.pdf]
